# Complete Genome Sequence of the γ-Hexachlorocyclohexane-Degrading Bacterium *Sphingomonas* sp. Strain MM-1

**DOI:** 10.1128/genomeA.00247-13

**Published:** 2013-05-16

**Authors:** M. Tabata, Y. Ohtsubo, S. Ohhata, M. Tsuda, Y. Nagata

**Affiliations:** Graduate School of Life Sciences, Tohoku University, Sendai, Japan

## Abstract

γ-Hexachlorocyclohexane (γ-HCH) is a man-made chlorinated insecticide that has caused serious environmental problems. Here, we report the complete genome sequence of the γ-HCH-degrading bacterium *Sphingomonas* sp. strain MM-1, which consists of one chromosome and five plasmids. All the specific *lin* genes that are almost identical to those of *Sphingobium japonicum* UT26 for the conversion of γ-HCH to β-ketoadipate are dispersed on four out of the five plasmids.

## GENOME ANNOUNCEMENT

γ-Hexachlorocyclohexane (γ**-**HCH) is a completely man-made chlorinated insecticide that has caused serious environmental problems due to its toxicity and long persistence in upland soils (1, 2). Our previous determination of the complete genome sequence of the archetypal γ-HCH-degrading alphaproteobacterium, *Sphingobium japonicum* UT26, revealed that its specific *lin* genes for the conversion of γ-HCH to β-ketoadipate are dispersed on chromosome 1 (*linA*, *linB*, and *linC*), chromosome 2 (*linF*), and pCHQ1 (a *linRED* cluster) (3). Other phylogenetically distinct γ-HCH-degrading sphingomonad strains carry almost identical specific *lin* genes, suggesting the spread of such genes by horizontal gene transfer (4).

To gain more insight into the bacterial adaptation mechanisms toward γ**-**HCH, the complete genome sequence of another γ-HCH-degrading bacterium, *Sphingomonas* sp. strain MM-1, was determined. Strain MM-1 was isolated from contaminated soil with technical-HCH (a mixture of α-, β-, γ-, and δ-isomers of HCH) in India (5). A fragment library of the total DNA of MM-1 was constructed for 454 sequencing, and 656,156 reads were obtained. Illumina mate-pair sequencing data (11,298,171 reads) were also obtained by a commercial company. These reads were assembled using Newbler, which generated the initial draft sequence data consisting of 21 scaffolds and 324 contigs. To facilitate the finishing process, we used GenoFinisher and AceFileViewer (6). Annotation by the Prokaryotic Genome Annotation Pipeline (PGAAP; http://www.ncbi.nlm.nih.gov/) was manually curated by using a dedicated tool bundled with GenomeMatcher (7), as well as by consulting the Microbial Genome Annotation Pipeline (MiGAP) for auto annotation (http://www.migap.org/).

The MM-1 genome consists of one circular chromosome (4,054,833 bp, 67.2% G+C, 3,801 open reading frames [ORFs]) and five circular plasmids: pISP0 (275,840 bp, 63.5% G+C, 251 ORFs), pISP1 (172,140 bp, 62.5% G+C, 174 ORFs), pISP2 (53,841 bp, 62.9% G+C, 51 ORFs), pISP3 (43,776 bp, 63.3% G+C, 44 ORFs), and pISP4 (33,183 bp, 63.0% G+C, 39 ORFs). The chromosome carries 55 tRNA genes and 2 rRNA gene operons. All the specific *lin* genes that are almost identical to those of UT26 are dispersed on four out of the five plasmids (*linF* on pISP0; *linA*, *linC*, and truncated *linF* on pISP1; *linRED* on pISP3; and *linB*, *linC*, and truncated *linF* on pISP4). The genome of MM-1 has 15 copies of IS*6100*, which is also highly associated with the specific *lin* genes in other strains, and all 15 copies are located on the five plasmids. Our sequence data clearly demonstrate that the genomic organization and localization of the specific *lin* genes of MM-1 are significantly different from those of UT26, suggesting the two strains have independently acquired the ability to utilize γ-HCH.

### Nucleotide sequence accession numbers.

The sequences with the annotation of MM-1 have been deposited in GenBank under accession no. CP004036, CP004037, CP004038, CP004039, CP004040, and CP004041 for chromosome, pISP0, pISP1, pISP2, pISP3, and pISP4, respectively.
